# Proposed Amendments to the Constitution of the International Commission on Zoological Nomenclature

**DOI:** 10.3897/zookeys.931.51583

**Published:** 2020-04-30

**Authors:** International Commission on Zoological Nomenclature

**Affiliations:** 1 c/o Lee Kong Chian Natural History Museum, 2 Conservatory Drive, Singapore 117377, Republic of Singapore International Commission on Zoological Nomenclature Singapore Singapore

## Abstract

The International Commission on Zoological Nomenclature (ICZN, or Commission) considered amendments to Articles of its Constitution (ICZN 1999a) at a special session in Singapore, convened on June 3–7, 2019. During this meeting, Commissioners also planned revisions to the Bylaws, the current International Code of Zoological Nomenclature (ICZN 1999b, 2003, 2012, 2017) and ZooBank user policies.

## Introduction

The International Commission on Zoological Nomenclature (ICZN, or Commission) considered amendments to Articles of its Constitution (ICZN 1999a) at a special session in Singapore, convened on June 3–7, 2019. During this meeting, Commissioners also planned revisions to the Bylaws, the current International Code of Zoological Nomenclature (ICZN 1999b, 2003, 2012, 2017) and ZooBank user policies.

The Commissioners voted to begin the process required to amend the Constitution. Notably, revisions were proposed and approved in principle by majority vote to clarify and update Constitutional Articles pertaining to:

1. Commissioners’ terms of service (Article 3);

2. inclusion of the ICZN web site as a required venue for information dissemination (Articles 2.1, 4.2 and 12.2);

3. outdated concepts (such as the ITZN, Article 13, or postal voting, Articles 4.6, 12.2, 16.1.4 and 16.1.5), and new responsibilities (such as Zoobank, Article 14.5);

4. duration of votes on amendments to the Code and Constitution (Article 16.1.4); and

5. separation of Procedures of the Commission from its Bylaws (Article 17).

Some Constitutional Articles were also proposed to be revised for consistency and accuracy, including:

1. Article 2.1 (“contact information” instead of “addresses”);

2. Article 4.1.1 (“countries of residence” instead of “nationalities”); and

3. adding “Executive” before “Secretary” in Articles 5.2, 7.1 and 14 to bring these Articles into accord with Articles 4.3, 5.3 and 9.

## Procedure for amendment

The Constitution of the ICZN (Article 12.2) requires publication of notice of a proposed amendment of the Constitution in the *Bulletin of Zoological Nomenclature* (*BZN*), and submission to at least three other journals, for comment from the zoological community for at least one year prior to the Commissioners’ voting on the proposed amendment (Article 16.1.2). The one-year period for comment on the current proposal, prior to any vote, will start on 30 April 2020, with the publication of notice in the *Bulletin of Zoological Nomenclature*. A copy of the proposed amendment will also be posted on the ICZN web site (www.iczn.org).

The ICZN requests comments from the community of zoologists on various parts of this amendment, either opposing, supporting, or suggesting improvements and alternatives. The Commission will revise the amendment in light of comments received from zoologists (Constitution Article 16.1.4). Comments that have been vetted for content and language will be published in *BZN* with open access and posted on the ICZN web site (www.iczn.org).

Formal comments should be sent to Dr Gwynne Lim, Executive Secretary of the ICZN (iczn@nus.edu.sg). Zoologists may also contact ICZN Commissioners directly for informal discussions (https://www.iczn.org/about-the-iczn/commissioners/). Assuming that sufficient consensus is reached in the zoological community, the final-worded amendment will be presented to the International Union of Biological Sciences (IUBS) for provisional ratification (Constitution Article 16.1.5.1). Effective ratification will be contingent on a subsequent vote by the Commission (Constitution Article 16.1.5.1). The decision and date of effective ratification will be published in *BZN* (Constitution Article 16.1.6).

## Proposed amendment

In the proposed amendment, normal font represents existing text of the Constitution that is retained. Strikethrough text is existing text that is to be deleted in the amended Articles. Underlined text is new text. Indented text in square brackets describes the changes. The amendment affects Articles 2, 3, 4, 5, 7, 9, 12, 13, 14, 16, and 17.

## Article 2. Membership of the Commission.

[Article 2.1 is to be revised to specify “contact details” instead of “addresses” and to include the ICZN web site as a source of information]

## 2.1. Number.

The Commission shall ordinarily consist of 18 members or such larger number as the Commission may decide. The names and contact detailsaddresses of the members at any given time shall be published in the *Bulletin of Zoological Nomenclature*and on the Commission web site.

[Articles 2.2 and 2.3 are unchanged.]

[Article 3 is to be revised to include the criteria for eligibility of re-election specified by Article 3.2]

## Article 3. Term of service and eligibility of members of the Commission.

[Article 3.1 is to be reformulated to simplify the rules for Commissioners’ terms of service and to eliminate the class system. Articles 3.1.1, 3.1.2 and 3.1.3 are to be deleted, and elements of Article 3.2 are to be simplified and merged into revised Articles 3.1.1 (length of term of service) and 3.1.2 (term of service for the President of the Commission).]

## 3.1. Normal Term of service.

3.1.1. The normal term of service of a member of the Commission shall be reckoned as follows:is eighteen years.

3.1.2. The term of service of the President of the Commission shall end at the end of the term indicated in the Bylaws even if it exceeds the eighteen years term of service above.

[Article 3.2 is to be revised to simplify the rules for re-election of existing Commissioners. Article 3.2.1 is to be deleted, having been subsumed within revised Article 3.1.1. Article 3.2.2 is to be reformulated as part of revised Article 3.2.1. Article 3.2.2.1 is to be reformulated as part of revised Article 3.2.2. Article 3.2.3 is to be deleted.]

## 3.2. Maximum term of service and Eligibility for re-election.

A member whose normal term of service has terminatedterminates may be re-elected but:

3.2.1. on completion of the period specified in Article 3.1 three years must elapse before a former member of the Commission is eligible for re-election;

3.2.2. this provision [Art. 3.2.1] shall not apply when a retiring or former member is pre-elected by the Commission to continue as or to become its President if re-elected as a member.

[Article 3.3 is to be revised to exclude Article 3.3.1, which is to be deleted. Article 3.3.2 is to be renumbered as Article 3.3.1. Article 3.3.3 is to be revised and renumbered as Article 3.3.2]

## 3.3. Prior termination of membership.

The membership of any member of the Commission shall terminate prior to the expiration of the term of service under art. 3.1 above:

3.3.1. on acceptance by the Council of notice of resignation tendered in writing to the Executive Secretary;

3.3.2.. if, not being on leave of absence, he or she fails on five consecutive occasions to return the ballot, or, when no ballot is provided, fails to record a vote for or against or an abstentionto record a vote or an abstention on questions put to the Commission for decision, provided that within a period of three months following such failure no written explanation has been made which the Council finds adequate.

## Article 4. Election of members of the Commission.

[Article 4.1.1 is to be revised to specify “countries of residence” instead of “nationalities” in recognition of the fact that some Commissioners are expatriates. Article 4.1.2. is to be revised to specify “refers” instead of “quotes”.]

## 4.1. Notice.

The Commission shall publish, not less than one year before a general session of the Commission [Art. 11.1], a notice which:

4.1.1. gives the names, countries of residencenationalities and fields of specialization of the members whose terms of service will end at the close of that session in accordance with Article 3;

4.1.2. quotesrefers to Article 2.2 and invites nominations for membership of the Commission;

4.1.3. gives a date, not more than three months before the forthcoming general session, by which nominations must be received.

[Article 4.2 is to be revised to include web sites as, depending on circumstances, either mandated or optional outlets for information]

## 4.2. Circulation.

The notice specified in Article 4.1 shall be published on the Commission web site and submitted to IUBS (or to its successor body, if any), to the organizers of the Congress where the general session is to be held, and to appropriate journals and/or web sites in different parts of the world, with a request for its dissemination.

[Article 4.3 is revised to add “Executive” before “Secretary” for consistency.]

## 4.3. Nominations.

Nominations, accompanied by a statement of the fields of specialization and qualification under Article 2.2 of each nominee, are to be sent to the Executive Secretary of the Commission. Unless the nomination contains the information, the Executive Secretary shall require each nominee to give consent to the nomination and to provide a curriculum vitae, a list of publications and a statement of his or her nomenclatural experience.

[Article 4.6 is to be revised to delete “postal”.]

## 4.6. By-elections.

The Commission may by a postal ballot fill vacancies arising from prior termination of membership [Art. 3.3], or which have not been filled by election at a session of the Section of Zoological Nomenclature [Art. 4.4.1], or which result from an increase in the number of members decided by the Commission in accordance with Article 2.1.

[Articles 5.2 and 5.3 are to be revised to add “Executive” before “Secretary”]

## 5.2. Between sessions.

It shall be the duty of a member of the Commission to vote, within the prescribed period, upon each question submitted to him or her for that purpose by the Executive Secretary.

## 5.3. Leave of absence.

A member of the Commission who is temporarily unable to perform his or her duties should apply through the Executive Secretary (if possible in advance) for leave of absence for a specified period.

[Article 6 is to be unchanged. Article 7.1 is to be revised to include “Executive” before “Secretary” and to include “or she” after “he”.]

## 7.1. The Executive Secretary to the Commission is also the Secretary to the Council but neither he or she nor any other member of the Secretariat shall vote in its deliberations.

[Article 8 is to be unchanged. Article 9 is to be revised to remove references to a Secretary-General and the International Trust for Zoological Nomenclature. The appointment of the Secretary-General is to be covered more generally in revised Article 14.]

## Article 9. Secretariat.

The Council may appoint an Executive Secretary for such a term and with such duties as may be fixed in the Bylaws. A member of the Commission may be appointed similarly as Secretary-General. The Executive Secretary may be an employee of an appropriate body such as the International Trust for Zoological Nomenclature.

[Articles 10 and 11 are to be unchanged. Article 12.2 is to be revised to remove “postal”, to include the Commission web site and to remove the necessity of submitting proposed amendments to the Constitution to multiple journals before voting on them. Article 12.2 is also to be revised to reduce the voting period from three months to two, in recognition of the faster transmission speed of electronic mail compared to postal mail.]

## 12.2. In cases involving the use of the plenary power or amendments to the Code or Constitution.

In such cases (see Articles 78 to 81 of the Code for the use of the plenary power and Article 16 of this Constitution for amendments to the Code or Constitution) an affirmative decision shall be deemed to have been taken only when two thirds of the votes validly cast in a postal vote lasting threetwo months are in favour of the proposal, and provided that notice of the proposal had been published in the *Bulletin of Zoological Nomenclature*, on the Commission web site and, only for the amendments to the Code, submitted for publication to at least two appropriate journals at least six months (in the case of amendments to the Code or Constitution, twelve months) prior to the vote.

[Articles 13 is to be revised to remove the reference to the International Trust for Zoological Nomenclature.]

## Article 13. Financial arrangements.

The Commission when not prepared to raise or administer its own funds is empowered for such purposes to enter into a beneficent relationship with one or more bodies such as the International Trust for Zoological Nomenclature, that undertake to act in accordance with the policy of the Commission and IUBS (or its successor body, if any). The Commission may terminate such a relationship at its discretion.

[Articles 14 is to be revised to include “Executive” before “Secretary” and to incorporate elements from Article 9.]

## Article 14. Editorial duties of the Commission.

The Commission shall issue and, finances permitting, may itself publish various communications, to be prepared and edited in the office of the Executive Secretary, or by another person appointed for that purpose, under the guidance of the Council.

[Articles 14.3 is to be revised to delete redundant language.]

## 14.3. Maintenance of*Official Lists* and *Indexes*.

The Commission shall compile and maintain the undermentioned *Lists* and *Indexes*:

14.3.1. *Official List of Family-Group Names in Zoology*;

14.3.2. *Official List of Generic Names in Zoology*;

14.3.3. *Official List of Specific Names in Zoology*;

14.3.4. *Official Index of Rejected and Invalid Family-Group Names in Zoology*;

14.3.5. *Official Index of Rejected and Invalid Generic Names in Zoology*;

14.3.6. *Official Index of Rejected and Invalid Specific Names in Zoology; Constitution*

14.3.7. *Official List of Works Approved as Available for Zoological Nomenclature*;

14.3.8. *Official Index of Rejected and Invalid Works in Zoological Nomenclature.*

[A new Article 14.5 is to be added to specify the Commission’s duties with regards to the *Official Register of Zoological Nomenclature*.]

## 14.5. *Official Register of Zoological Nomenclature* (ZooBank).

The Commission shall maintain ZooBank, the*Official Register of Zoological Nomenclature* .

[Article 15 is to be unchanged. Article 16.1.2 is to be revised to specify the time span as “twelve months” instead of “one year”. Articles 16.1.4 and 16.1.5 are to be revised to remove “postal”. Article 16.1.4 is to be revised to reduce the voting period from three months to two, in recognition of the faster transmission speed of electronic mail compared to postal mail.]

16.1.2. receive and consider comments from zoologists that are received within twelve monthsone year of the publication of the proposals;

16.1.4. vote on the proposed amendments (which may be modified in the light of the comments by zoologists and the Section) in a postal vote lasting twothree months [Art. 12.2];

16.1.5. submit the amendments subject to their approval by two thirds of the votes validly cast in the postal vote, and with the support of the Section for the major principles, to IUBS (or to its successor body, if any) for ratification [Art. 90 of the Code];

[Articles 17 is to be revised to distinguish between Bylaws and Procedures.]

## Article 17. Bylaws and Procedures.

The Commission is empowered to adopt a set of Bylaws and of Procedures governing those of its regulations and activities not covered by the Constitution.

The Commission has authority to modify these Bylaws and Procedures by a majority vote as the occasion demands. These Bylaws will deal with such matters as the duties of the Officers, the methods by which nominations are to be obtained for vacancies on the Commission, the relations between the Commission and the Secretariat, and with other business matters of the Commission. The Procedures will deal with regulations concerning the treatment to be given to applications and the adoption of time schedules and priorities, and with other business matters of the Commission.

[Article 18 is to be unchanged.]
